# Molecular characterization of high-risk humanpapillomavirus genotypes in women with or without cervical lesions at VIA/VILI in Kara, Togo

**DOI:** 10.4314/ahs.v21i4.27

**Published:** 2021-12

**Authors:** Essolakina Dolou, Akouélé Kuassi-Kpede, Theodora M Zohoncon, Ina Marie Traore, Gnatoulma Katawa, Alice R Ouedraogo, Esther Mah Traore, Prosper Bado, Clarisse T Ouedraogo, Florencia Djigma, Abdoul-Samadou Aboubakari, Simplice Damintoti Karou, Jacques Simpore

**Affiliations:** 1 Pietro Annigoni Biomolecular Research Center (Cerba / Labiogene), Pr Joseph KI-Zerbo University, Burkina Faso; 2 Biological and Food Techniques School (ESTBA-UL), University of Lome, Togo; 3 Obstetrics Gynecology Department, Kara University Hospital Center, Kara, Togo

**Keywords:** Human papillomavirus, High-risk, Genotyping, Kara, Togo

## Abstract

**Background:**

Persistent infection with high-risk (HR) papillomavirus (HPV) genotypes plays a central role in the pathogenesis of invasive cervical cancer.

**Objectives:**

This study aimed to determine the prevalence and distribution of HR-HPV among women with or without cervical lesions at VIA/VILI in Togo.

**Methods:**

Cervical samples were collected from 238 women with or without cervical lesions at VIA / VILI and[c3] DNA [c4]was extracted and analyzed by real-time multiplex PCR. Logistic regression analysis was used to determined risk factors associated with HPV infection.

inPietro Annigoni Biomolecular Research Center (CERBA / LABIOGENE) in Burkina Faso.

**Results:**

The age of the women ranged from 17 to 61 years old, and most were married (73.5%). The prevalence of HRHPV was 35.71% and this was higher in the age range 35–39 years. The six most common genotypes were HPV 31 (18.7%), HPV 52 (13.82%), HPV 68 (13.01%), HPV 66 (9.76%), HPV 58 (8.13%) and HPV 56 (8.13%). Genotypes HPV 18 (4.07%) and HPV 16 (0.81%) were less frequent.[c5] Married or living with a partner was associated with HPV infection (OR=2,17, IC [1.20–3.91], p<0,009).

**Conclusion:**

This study allowed characterizing for the first time in Togo, HR-HPV genotypes. This will help mapping-HR-HPV genotypes circulating in West Africa.

## Introduction

Humanpapillomavirus infection is a necessary (but not sufficient) risk foctor [c6]of cervical cancer^1^. According to GLOBOCAN 2018, cervical cancer was the fourth most common diagnosed and the fourth leading cause of cancer death in women, with 570,000 cases and 311,000 deaths worldwide in 2018^2^. One of the most important intervention to control these infections is vaccination^3^. Since HPV genotypes prevalence and distribution differ among regions^4^, ^5^, molecular characterization of these genotypes in each region of the world are necessary in order to provide background data to guide the choice of an appropriate vaccine for each populations. However, molecular epidemiology of HR-HPV infection in women in Togo remains understudied. The aim of this study was to determine by real-time multiplex PCR the prevalence and distribution of high-risk HPV genotypes among women with or without cervical lesions at VIA/VILI in Togo, in order to contribute to West Africa mapping.

## Materials and Methods

This population based cross-sectional study was conducted in women with or wthout cervical lesions aged between 17 and 61 years from Kara in north Togo. All women gave their written informed consent before participating in the study. For minors (under 18 years old), the consent of a parent or tutor was required in additional to their own informed consent. From December 27, 2016 to January 06, 2017, we recruited238 women by random sampling after mass sensitization and those coming for gynecology consultation at the gynecology department of the «Hippocrates cabinet». The virgin or pregnant women or during menstrual period or having undergone total hysterectomy were not included in thisstudy. Midwifes administered a questionnaire to each woman in order to get information on their socio-demographiccharacteristics and sexual behavior. Endocervical samples were collected by gynecologist by swabbing. Samples was introduced into a transport medium (Transport medium from the Sacace kit) and stored at −20°C. At the end of collection, the frozen samples were introduced into coolers fitted with ice and carried to Pietro Annigoni Biomolecular Research Center (CERBA / LABIOGENE) in Burkina Faso.

sequentially or concurrently by visual inspection after acetic acid and Lugol applications (VIA/VILI).

DNA extraction was carried out using SACACE biotechnologies ® DNA-Sorb-A kit (Sacace Biotechnologies, Como, Italy). The main steps of extraction are: lysis of cells membranes, fixation of DNA on columns, washing and elution.

Lyse step: Add 300µl of lysis solution to 100µl of sample. Mix by vortexing, incubate at 65°C for 5min and centrifuge at 12000g for 10min. Transfer the supernatant into eppendorf tube.

DNA fixation step: Add 20µl of Sorbent, incubated for 3 min at room temperature (repeat the step). Centrifugate for 30 sec at 5000 g. Remove the supernatant with micropipette into another tube

Washing step: Add 500µl of washing solution. Vortex and centrifuge at 10000g for 30sec. Discard the supernatant (repeat this step). Dry the pellet for 5–10 min at 65°C

Elution step: Add 100µl of DNA-eluent. Incubate for 5 min at 65°C and vortex and centrifuge. Stored the supernatant at - 20°C for DNA amplification

The genotyping was performed by real-time multiplex PCR, using the SACACE biotechnologies® HPV Genotypes 14 Real-TM Quant V67-100 FRT kit, according to the manufacturer's instruction. For amplification, prepare 4 tubes for each clinical sample, 4 tubes for standards K1, 4 tubes for standards K2, 4 tubes for Negative control. The final reaction volume was 25µl containing: 10µl of specific primers (PCR-mix 1 “16,18,31,IC”, PRC-mix 2 “39,45,59,IC”, PRC-mix 3 “33,35,56,68”, PCR-mix 4 “51,52,58,66”), 5µl of mix-PCR-Buffer-FRT and DNA-polymerase, 10 µl of extracted DNA sample. For control and standards add, 10 µl of Negative control into control tube, 10 µl of each K1 et 10 µl of each K2 into standards tubes.

[c14]The data was processed and analyzed using Excel database, SPSS 21 and Graph Pad 6. The Chi-square test and the exact Fisher test were used to compare the proportions and univariate regression analysis were used to study the risk factor associated with HPV infection. [c15][D16]The p-value threshold for the Chisquare test and the exact Fisher test was 0.05 and 0.2 for the univariate regression analysis.

## Results

### Study population

Of a total of 238 women included in our study, 13 (5.46%) came for gynecological consultation purpose and 225 (94.54%) came only for cervical cancer screening after mass sensibilization. For those who came for gynecological consultation purpose, the main motifs of their consultation: was dysmenorrhea (1.7%); desire for a child (0.42%); leucorrhoea (0.8%); pruritus (0.42%); dysuria (0.42%); pelvic ultrasound (0.42%); vaginal infection (1.3%).

### Socio-demographic characteristics of women

As recorded in the table 1, the age of women ranged from 17 to 61 years with an average of 34.67 ± 9.44 years. The 35–39 age group was the most represented (22.3%). Women out of school accounted for 18.5%, compared to 55% who exceeded primary school. The majority (73.5%) of women were married.

**Table 1:** Socio-demographic characteristics of women

Characteristics	Number	(%)
**Age groups in years**		
≤24	32	13.4
25–29	42	17.6
30–34	44	18.6
35–39	53	22.3
40–44	31	13
≥45	36	15.1
**Education level**		
None	44	18.5
Primary	63	26.5
Secondary	89	37.4
University	42	17.6
**Marital status**		
Living alone (celibate, divorced, widowed)	63	26.5
Married	175	73.5
**Profession**		
Housewives	45	18.9
Traders	83	34.9
Officers	41	17.2
Craftswomen	44	18.5
Students	25	10.5

### Behavioral and sexual characteristics of women

As it is shown in table 2, the age at first intercourse reported by women ranged from 9 to 33 years with an average age of 18.50 ± 2.86 years. In the study 85.7% of women had their first sexual intercourse under 21 years old and; 96.1% have only one current sexual partner.

**Table 2:** Behavioral and sexual characteristics of women

Characteristics	Number	(%)
**Age at the first sexual** **intercourse**		
[9–20]	204	85.7
[21–33]	34	14.3
**Condom use**		
Never	129	56.1
Occasionally	91	39.6
Every time	10	4.3
**Number of current sexual** **partners**[c19]		
1	221	96.1
>1	9	3.9
**Number of pregnancies**[c20]		
None	33	13.9
1–4	153	64.3
≥ 5	52	21.8
**Parity**		
None	52	21.9
1	51	21.4
>1	135	56.7
**Cigarette** **consumption**[c21] **in lifetime**		
Yes	3	1.3
No	225	98.7
**Contraceptive** **use**[c22] **(current or past)**		
Yes	39	16.4
No	199	83.6
**Cervical cancer screening**[c23]		
Yes	9233	3.897.9
No	2295	96.22.1

### Prevalence of cervical lesions diagnosed with VIA / VILI

233 women out of 238 were screened at VIA/VILI. The raisons why the test was not performed in five other women was bleeding and discomfort. The prevalence of cervical lesions diagnosed by VIA and VILI were 0.86%.

### Prevalence of high risk genotypes

**Figure 1:**
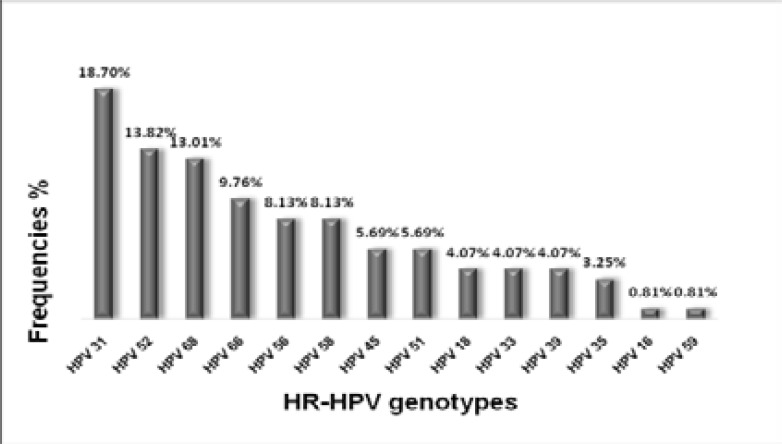
Prevalence of HR-HPV genotypes in women

In our study, all samples tested were positive for *β*-globin gene. The estimated HR-HPV prevalence among 238 women was 35.71% (85/238). As shown in figure 1, the six most common HR-HPV genotypes in our population were HPV31 (18.7%), HPV52 (13.82%), HPV68 (13.01%), HPV66 (9.76%), HPV58 (8.13%) and HPV56 (8.13%). The combined proportion of 7 HR-HPV (16/18/31/33/45/52/58) included in Gardasil-9 vaccine were 55%. Those (HR-HPV16/18) included in Gardasil-4andCervarixvaccines were detected together in a proportion of 5%. The genotypes found in women positive at VIA / VILI are HPV 35, 51, 52, 66. One association of HPV 35 and 52 was observed in the lesions..

### Analyses of proportions and risk factors associated with HR-HPV infection

The results in Table 3 show that the prevalence of HPV infection varies with the age. It increases with age up to 30 years and then decreases after 30 years. The most affected age groups are [25–30] and [40–44] with respectively 22.4% and 22.2%. The prevalence is higher among women living alone than the married women, 49.2% (31/63) versus 30.9% (54/175). The prevalence is 36.8% (75/175) among women who had their first intercourse before age 21 compared to 29.4% (10/34) but this difference is not statistically significant. Women who use condoms are more infected than those who do not use; 33.3% (43/129) versus 38.6% (39/101). The infection is lower among women with one sexual partner than those with more than one sexual partner, 34.4% (4/9) versus 44.4% (76/221). The proportion of infected women decreases with parity, from 42.3% (22/52) in nulliparous to 31.9% (43/135) in multiparous. The prevalence is almost identical among women using contraception and those without contraception, 35.9% (14/39) and 35.7% (71/199). HPV infection is more common in women who have never been screened for cervical cancer 36.2% (83/229) than those who have been tested at least once.

**Table 3:** Factors associated with HR-HPV infection

Characteristics	HPV+ n=85	HPV- n=153	Total n=238	OR	IC95%	p
**Age groups in years**						
<25	12	20	32			
25–29	19	23	42			
30–34	13	31	44	-	-	
35–39	12	41	53			0.019
40–44	18	13	31			
>44	11	25	36			
**Marital status**						
Single	31	32	63			
Married or living with a partner	54	121	175	2.17	1.20–3.91	**0.009**
**Age at the first** **sexual intercourse**						
≤20	75	129	80204	11.40	0.63–3.08	0.41
>20	10	24	34			
**Condom use**						
No	43	86	129	00.79	0.46–1.37	0.41
Yes	39	62	101			
**Number** **of current sexual** **partners**						
One	76	145	221	0.42	0.11–1.61	**0.29**
More than one	4	5	9			
**Parity**						
None	**22**	**30**	**52**			
One	**20**	**31**	**51**	-	-	**0.34**
More than one	**43**	**92**	**135**			
**Contraception** **use (current or past)**						
Yes	14	25	39	1.01	0.49–2.07	**0.98**
No	71	128	199			
**Cervical cancer** **screening**						
No	83	146	229	1.99	0.40–9.80	0.50
Yes	2	7	9			

Univariate logistic regression revealed that married or women living with a partner were more exposed to HPV infection than women living alone (OR=2,17, IC [1.20–3.91], p<0,009).

VIA/VILI and smoking did not allow analysis of associations with HR-HPV infection. Eight women did not give information about number of sexual partners and condom use.

## Discussion

This study which allow to identify HR-HPV among women in Kara region, is for our knowledge one of the first study in Togo. Even though our sample size is not sufficient to generalize the conclusions for all of the population of Togo, these results remain valid for study populations. The average age of the women in our study was 34.67 ± 9.44 years. The average age of women at first sexual intercourse was 18.50 ± 2.86 years. These results are similar to those of Traoré et al. who found 35.3 ± 8.1 years for the average age and 18.57 ± 2.2 years for mean age at first intercourse, in Bobo-Dioulasso^6^.

The prevalence of cervical lesions diagnosed with VIA / VILI was 0.86%. This prevalence is lower than that found in Bobo-Dioulasso (5.0%) by Traore et al.^6^. This difference may be due to the subjectivity of these tests, because the result depends on the observation.

The prevalence of HR-HPV infection among women in this study was high (35.71%) compared to the adjusted global HPV prevalence in western Africa region (17%) as reported by de Sanjose et al. 4. This high rate indicate that the transmission of the infection continues to increase, hence the importance of implementing prevention measures such as vaccination. Several studies have reported various prevalences. It is 33.2% in Benin, 25.4% in Bobo-Dioulasso^6^, 18.6% in Southwest Nigeria^8^, 41.5% in Ouagadougou^9^, and 32.1% in Conakry Guinea^10^. These variations in the reported rates may be justified by the age structure of different studies populations. The high prevalence reported by Ouedraogo et al. is justified by the youngest age of its population compare to ours. Compare to others authors, the proportion of women under 25 years in our study was higher than other authors by region, all studies agree that it is more frequent among women at younger ages. Moreover, the high risk of exposure of young women to multiple partners explain this high rate.

The six most common genotypes found in this study was, HPV 31 (18.7%), HPV 52 (13.82%), HPV 68 (13.01%), HPV 66 (9.76%), HPV 56 (8.13%) and HPV 58 (8.13%). Similar genotypes have been reported by many other authors, such as HPV39 and 52 in Bobo Dioulasso^6^; HPV35, 52, 31 in Ouagadougou11, HPV 52 in Conakry Guinea10. All women tested positive at VIA / VILI carry at least one HPV genotype.

**Figure 2:**
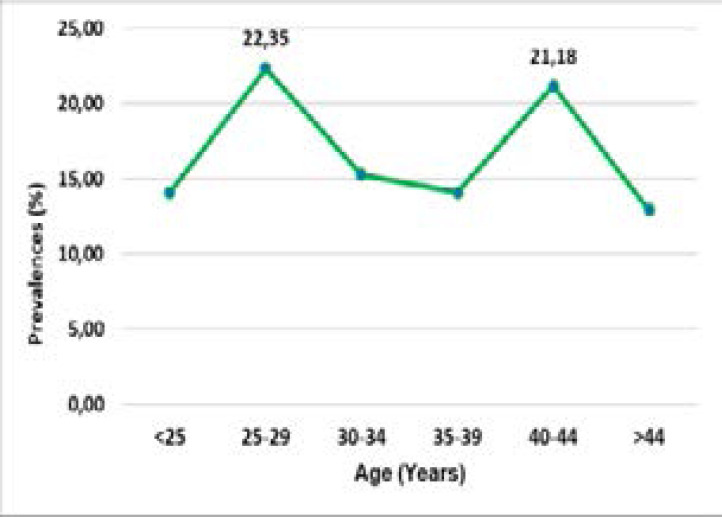
Prevalence of HR-HPV infection by age

According to age, the prevalence of HR-HPV infection showed two picks; the first (22.4%) between 25–29 years old and the second (21.2%) between 40–44 years old (figure 2). Sanjoseet al. found the first pick younger than 25 years and the second peak after 45 years^4^. The first peak confirms that HPV infections are most common in women at young age.The second pick can be explained by the reactivation of a latent infection following the alteration of the immune system or by changes in sexual behavior of women and their partners as suggested by Sanjose^4^. This result is different from that of de Sanjose. This difference is explained by the small size of our sample 238 versus 157 879.

Our study also reveals that the 7 HR-HPV genotypes included in the Gardasil-9 vaccine represent 55% whilethose included in Gardasil-4 and Cervarix represent only 5%.All these results suggest that both Gardasil-4 and Cervarix vaccines confer limited protection against HR-HPV infection in our population. However, the Gardasil-9 could be suitable for our population.

Several Many risk factors are reported to be associated to HR-HPV infection such as: age; marital status; condom use, age at first intercourse, number of sexual partners, parity, oral contraception; cervical cancer screening participation^14^. Ouédraogo et al. found in Burkina Faso a significant association between HR-HPV infection and marital status with p = 0.028 15. Our study found also that women living alone were 2.17 (table 3) fold more susceptible for HR-HPV infection than those who are married, suggesting their greater exposure to multiple partners.

## Conclusion

This study identified fourteen genotypes among women in Kara. The high prevalence of HPV infection shows that women are exposed continuously to high risk of transmission. The most common genotypes found in our study does not have vaccines available. The HPV16 and 18 genotypes that have vaccines are rare. These results provide new directions on the strategy to adopt to fight against cervical cancer in this population and also direct the choice of the suitable vaccine for our populations. Although the study has shown that the most common genotypes are other than genotypes 16 and 18, the role that play these genotypes in the development of cervical neoplasia and cancer remains unknown.
